# Recurrence of a Langerhans Cell Histiocytosis bone lesion in a different site: A case report

**DOI:** 10.1016/j.amsu.2022.103401

**Published:** 2022-02-24

**Authors:** Ali Al Abdulsalam, Kusum Kapila, Mohammad Alherz, Mohammad Alsayegh

**Affiliations:** a Department of Radiology, Mubarak Al-Kabeer Hospital, Kuwait; b Department of Pathology, Mubarak Al-Kabeer Hospital, Kuwait

**Keywords:** Langerhans' cell histiocytosis, Recurrence, Sternum, Neoplasia, Pelvis, Case report

## Abstract

**Introduction:**

Langerhans’ Cell Histiocytosis is a rare disease of unknown etiology, the pathogenesis of which involves both reactive and neoplastic processes. Despite potential resolution with conservative management, a rare recurrence in a distant site after 3 years from presentation in this case highlights the variability in the course of the disease and the need for larger studies to enable recognition and evidence-based management.

**Case presentation:**

We present an unusual case below of a 25-year-old gentleman who presented with sternal pain and tenderness. Imaging showed a lytic lesion in the sternum which resolved spontaneously with oral analgesia. He presented 3 years later with back pain and a similar lytic lesion in the iliac bone was found and diagnosed as recurrent Langerhans Cell Histiocytosis with a biopsy.

**Clinical discussion:**

There is a diverse array of documented presentations of Langerhans Cell Histiocytosis involving single or multiple systems, while its progression and outcomes are equally unpredictable from the current literature. In order to facilitate wider recognition, Langerhans Cell Histiocytosis should be considered in the differential diagnosis of recurrent lytic bone lesions.

**Conclusion:**

Although this is a rare disease, there is currently an unmet need for understanding the determinants of recurrence and response to treatment.

## Highlights

• Langerhans Cell Histiocytosis is a rare disease of reactive and neoplastic pathologies.
• Spontaneous remissions can be followed by recurrences in distant sites.
• It should be considered in the differential diagnosis of recurring lytic bone lesions.

## 1 Introduction

Langerhans Cell Histiocytosis (LCH) is a rare idiopathic condition characterized by a neoplastic process and an abnormal reactive process. The resulting local or systemic effects can involve multiple systems in the body, most commonly the skin, lymph nodes, central nervous system, bone and lungs [1]. Although it can present at any age, it is more common in the pediatric age group [2]. We present an unusual case below of a 25-year-old gentleman with spontaneous remission of a sternal LCH lesion, recurring in the pelvis after 3 years from initial presentation. The case is presented in line with the SCARE criteria [3].

## 2 Case report

**Fig. 1:**
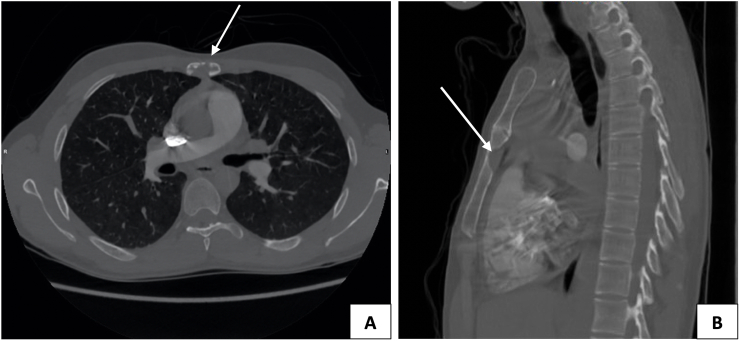
(A) Axial and (B) Saggital CT chest with a lytic lesion of the sternum in bone window.

A previously healthy 25-year-old Middle Eastern male self-presented to our general hospital three years ago with sternal chest pain of a gradual onset, without any associated symptoms. He had no family history of inheritable conditions and no history of drugs or allergies. The patient was a non-smoker without any alcohol intake. On physical examination his vital signs were within the normal range and sternal tenderness was elicited on palpation. No other sites of pain were reported, and review of systems was unremarkable. A posterior-anterior (PA) chest radiograph showed no abnormalities. A computed tomography (CT) scan of the chest was performed, which showed a lytic bone lesion in the sternum (Fig. 1A and B). Biopsy of the lesion was inconclusive. Opting to receive only oral analgesics (Paracetamol, Ibuprofen) as needed for the pain following a discussion of the existing management options, the lesion resolved spontaneously within a year with no deviations from the initial management plan. The patient presented twice in clinic for follow-up since his initial presentation. He described a gradually decreasing need for analgesia which was ceased after 3 months, with no impact on his quality of life. Routine labs remained within normal range and no complications were observed. He was subsequently lost to follow-up.

**Fig. 2:**
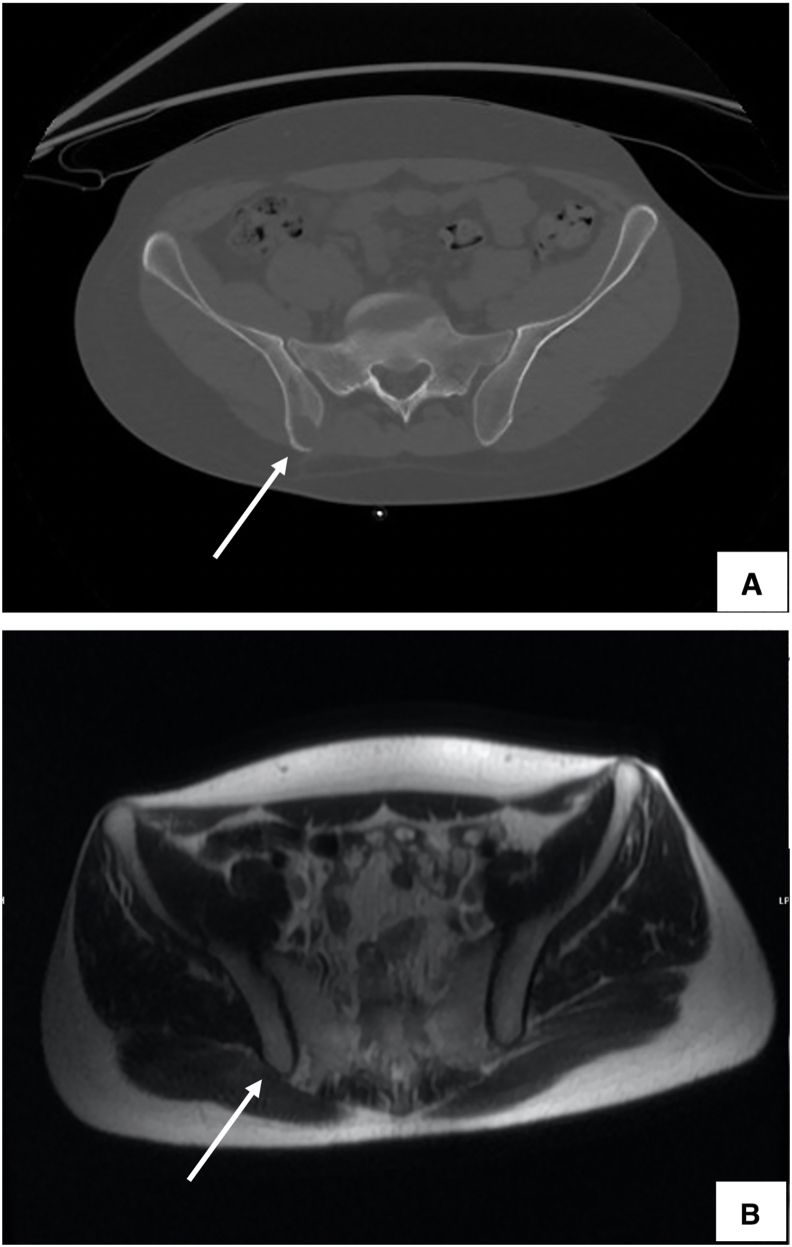
(A) Axial CT pelvis with bone showing a new lytic lesion of the right posterior inferior iliac spine. (B) MRI pelvis with axial SS-FSE sequence 3 years previously showing normal signal intensity in both iliac bones.

He presented again 3 years later with a 1-month history of lower back pain without neurological deficits or other symptoms. Basic laboratory tests including a complete blood count, renal function tests, and liver function tests were within normal range. Plain radiographs of the lumbar spine and pelvis were unremarkable. Given his history, a CT scan of the pelvis was obtained which revealed a lytic bone lesion of the right posterior inferior iliac spine (Fig. 2A) which was not evident in magnetic resonance imaging (MRI) with a single shot fast spin echo (SS-FSE) sequence that was performed 3 years previously (Fig. 2B).

**Fig. 3:**
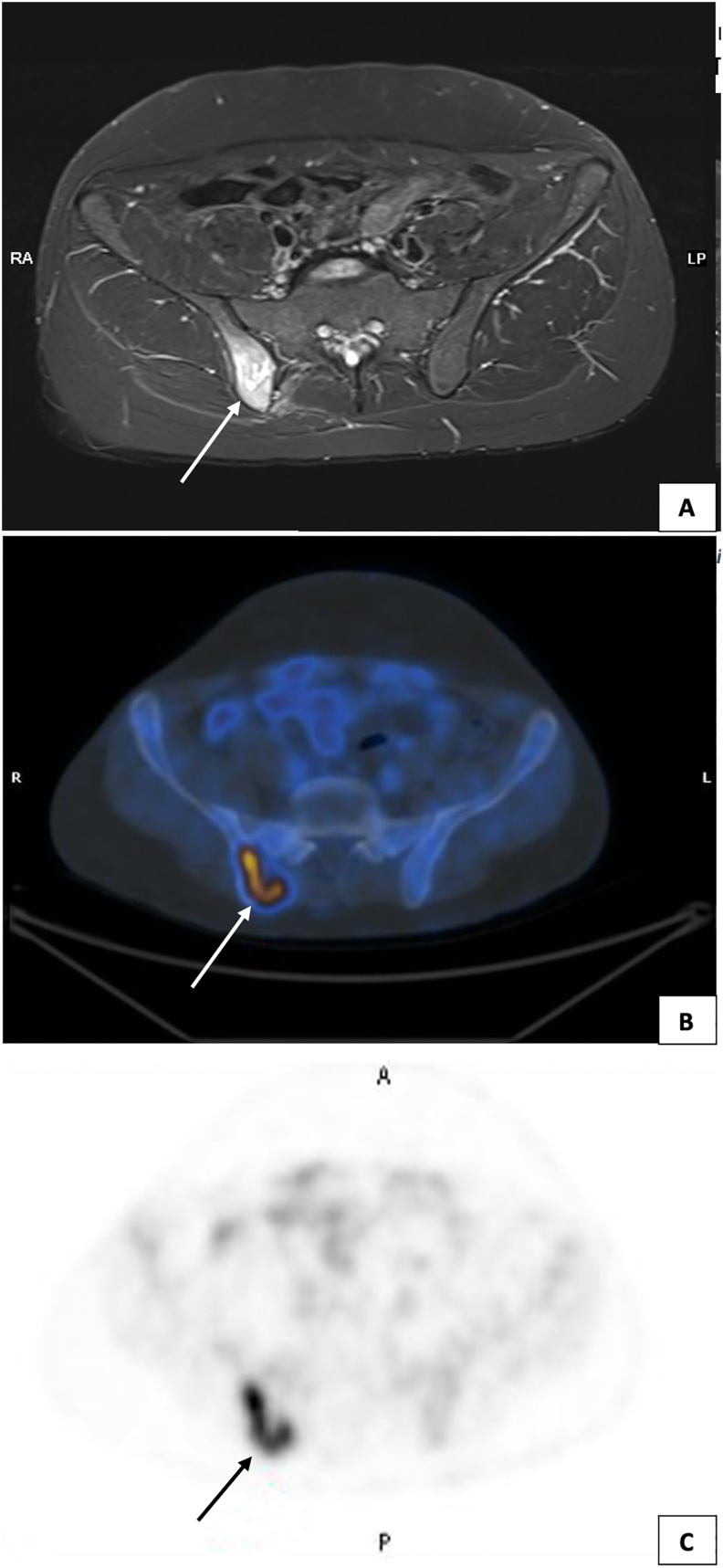
(A) MRI Pelvis with T2 STIR sequence showing increased signal intensity in the right posterior inferior iliac spine. (B–C) PET CT scan showing uptake of 18-FDG.

Further evaluation with an MRI scan was conducted, which revealed an increased signal intensity in the right posterior inferior iliac spine on T2 STIR sequence (Fig. 3A) The patient then underwent positron emission tomography (PET)/CT to evaluate the lesion and look for concurrent lesions. The PET CT scan confirmed a solitary suspicious lesion which required further evaluation with a biopsy (Fig. 3B and C). He underwent CT guided fine needle aspiration cytology (FNAC) by the interventional radiologist, and the samples were sent for histopathology evaluation.

**Fig. 4:**
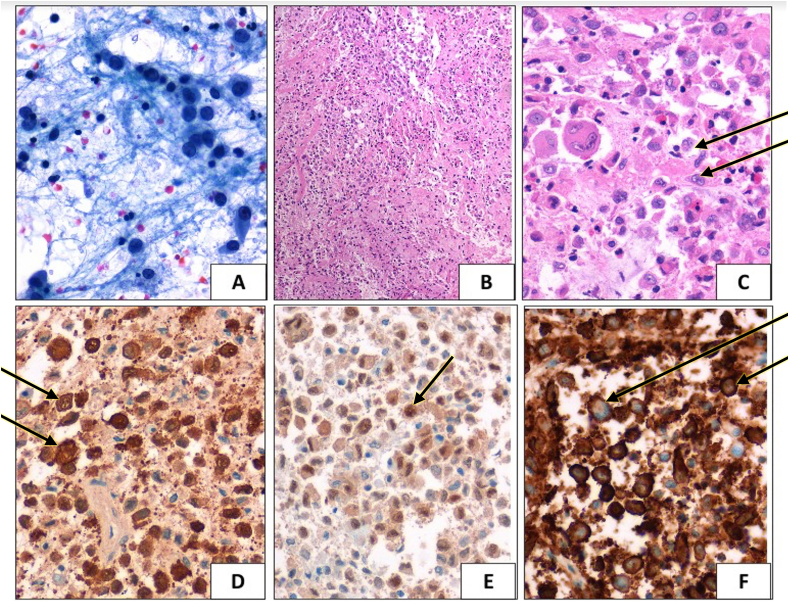
Cytological specimen (A–C) featuring moderate cellularity with numerous large mononuclear Langerhans cells admixed with lymphocytes, plasma cells, eosinophils and giant cells. (C) shows multiple grooved and folded nuclei {Papanicolaou × 400; Hematoxylin Eosin [H&E] × 200; [H&E] × 200}. Immunocytochemistry shows positivity of Langerhans cells for S100 with nuclear and cytoplasmic expression (D), Cyclin1 (E) and CD1a membrane positivity (F).

The histopathologic examination revealed large histiocytes with irregular, vesicular, grooved and folded nuclei with a dense infiltrate of eosinophils. Occasional giant histiocytic cells were also seen (Fig. 4C). Immunohistochemical staining was positive for CD1a, S100 and cyclin D1 (Fig. 4D–F). A diagnosis of Langerhans’ Cell Histiocytosis (LCH) was made.

## 3 Discussion

We present a case of a previously healthy 25-year-old man who presented initially with a sternal lytic lesion which resolved within a year and recurred in the right posterior inferior iliac spine which was confirmed by the histopathology examination as LCH. This rare presentation of LCH is worthy of discussion.

Featuring an uncontrolled proliferation of histiocytes, Histiocytosis comprises a range of diseases which present with single or multisystem involvement, including skin, central nervous system, lung, gastrointestinal tract, and bone [4]. Based on cell lineage, histiocytosis can further be classified into Langerhans and non-Langerhans histiocytosis. Previously considered specific to epidermal Langerhans cells, the discovery of Birbeck granules in what was formerly known as Histiocytosis X precipitated the entity's name change [5]. Studies have since shown that cells from LCH are similar to dendritic cells that originate from the bone marrow as opposed to epidermal Langerhans cells [6]. The pathologic manifestations of LCH can be classified into two main processes [7]. Firstly, an abnormal reactive process is indicated by the spontaneous remissions, good survival in single lesions and the involvement of multiple cytokines. Secondly, a neoplastic process is characterized by the multisystem involvement with increased mortality and response to chemotherapy [8]. In addition, the recent association with the BRAF signaling gene mutation (V600E) found in 57% of LCH lesions, supports the neoplastic aspect of the disease [9]. As such, LCH is now considered as an inflammatory myeloid neoplasm.

LCH can present at any age group but is most commonly encountered in children aged less than 15 years old, when the incidence is estimated to be 3–5 cases per million per year [2]. Whereas in all adult age groups, the incidence is 1–2 cases per million per year [10], making an onset at 25 years in this case an even rarer presentation. The case is further distinguishable by the rarity of the location in the pelvis. In a systematic review of 64 cases of LCH published over a 19 year period, the most affected bone sites were the skull (42.2%) and chest wall (23.4%) followed by the spine and pelvis (9.4%) [1]. In our case the patient initially presented with a chest wall lesion, and then had a recurrence in the pelvis. According to the literature, chest wall lesions are common sites, whereas lesions in the pelvis are rare [1].

Though the incidence of remission has not been formally quantified in the literature, the highest rates are generally seen in unifocal disease, with documented remissions of bone lesions occurring in sites such as the femur, orbit, intracranial bones, maxilla and mandible [11,12]. Recurrences have also been observed, but rarely in a different site and usually within 12–18 months after initial diagnosis [13]. Similar to the presented case, a recurrence of LCH in different sites was reported after 16 years with initial sacroiliac involvement subsequently presenting as an orbital lesion [14].

## 4 Conclusion

Spontaneous remission of LCH is a previously documented phenomenon. However, there is possibility for recurrence of single bone lesions in the same site or a different site as in our case. Although LCH is considered a rare disease, it should be included in the differential diagnosis for a recurring lytic bone lesion. In addition, familiarity with the radiological findings of LCH is vital in evaluating the potential multisystem involvement of the disease.

## Provenance and peer review

Not commissioned, externally peer-reviewed.

## Sources of funding

None.

## Ethical approval

The study type is exempted from ethical approval at our institution: case report.

## Consent

Written informed consent was obtained from the patient for publication of this case report and accompanying images. A copy of the written consent is available for review by the Editor-in-Chief of this journal on request.

## Author contribution

AA: conducted literature review, drafted the manuscript. KK: Provided histopathology slides and diagnosis. MH: Editing and revision of the manuscript. MS: conceptualised and critically revised the manuscript. All authors approved the final manuscript as submitted.

## Registration of research studies

Not Applicable.

## Guarantor

Mohammad Alsayegh.

## Declaration of competing interest

None.
